# The unknown biogeochemical impacts of drying rivers and streams

**DOI:** 10.1038/s41467-022-34903-4

**Published:** 2022-11-24

**Authors:** Margaret A. Zimmer, Amy J. Burgin, Kendra Kaiser, Jacob Hosen

**Affiliations:** 1 Department of Earth and Planetary Sciences, University of California, Santa Cruz, Santa Cruz, CA 95064 US; 2 University of Kansas and Kansas Biological Survey-Center for Ecological Research, Lawrence, KS 66047 US; 3 Department of Geosciences, Boise State University, Boise, Idaho 83725 US; 4 Department of Forestry & Natural Resources, Purdue University, West Lafayette, IN 47907 US

**Keywords:** Hydrology, Biogeochemistry

## Abstract

Rivers and streams are increasingly drying with climate change and biogeochemical impacts may be important. In this comment the authors discuss the challenges to the biogeochemistry of non-perennial rivers and streams, and what can be done to tackle them.

## Teaser

Non-perennial rivers and streams - those that periodically cease flowing - are critical components of aquatic systems and comprise over half of global river and stream systems. We argue for coordinated, collaborative, standardized, and open efforts to understand their unique biogeochemical behaviour, which is becoming ever more pressing due to pronounced shifts between wet and dry as the climate changes.

**Fig. 1:**
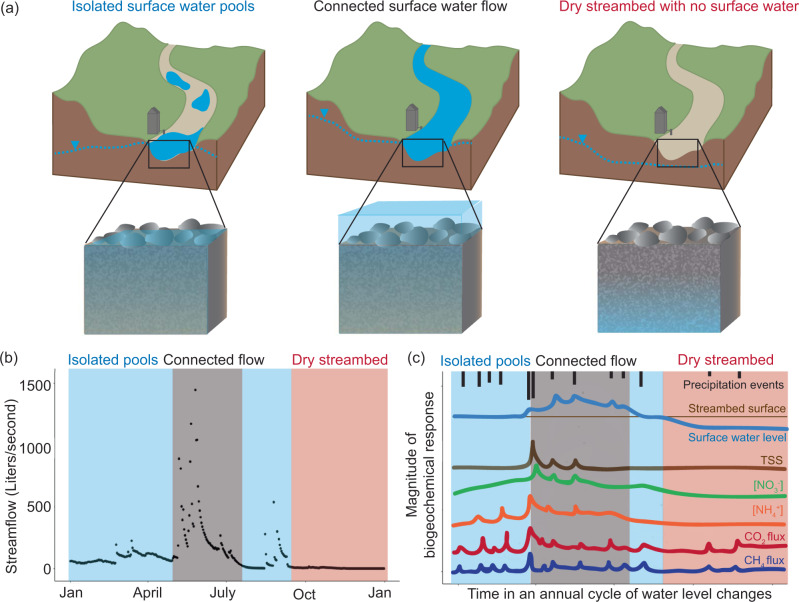
Biogeochemical response examples to different in-stream hydrologic conditions. **a** Illustrations of reach scale and streambed hydrologic conditions during periods with (left to right): isolated surface water pools, connected surface flow, and a dry stream with no surface water. **b** Example streamflow hydrograph with periods of isolated surface water pools (blue), connected surface water flow (gray), and dry streambed (red). Streamflow data from Konza Prairie Biological Station. **c** Hypothesized chemical responses to fluctuations in surface water levels relative to streambed surface elevation. The illustrated surface water level time series is simplified replication of streamflow data from panel b. TSS = total suspended sediment, [NO_3_−] and [NH_4_^+^] = nitrate and ammonium concentration in surface water, CO_2_ and CH_4_ flux = flux as the transfer from the stream channel to atmosphere.

Rivers, encompassing all flowing waters including streams, are dynamic and important features in our landscapes, transporting sediment, carbon, and other materials to our oceans, contributing to global nutrient cycles, and fulfilling critical economic and resource needs. Yet, over half of global river networks periodically cease flowing^1^. These drying rivers are colloquially referred to as non-perennial or intermittent rivers. Such non-perennial systems are sites of dynamic biogeochemical processes and microbial communities as their wetness states shift^2,3^. Biogeochemistry ultimately determines the quality of water, which determines what water can be used by humans for drinking water, recreation, and agriculture. Non-perennial rivers transport considerable amounts of sediment and nutrients when flow re-activates, which can significantly impact downstream water quality and ecosystem processes (e.g., nutrient removal)^4^. For example, sediment transport is a natural process of river networks, but excess nutrients bound to sediment can decrease water quality (Fig. 1). Thus, understanding how changes in streamflow, including periods of complete dryness, will impact processes like sediment transport has implications for water quality at a world-wide scale.

Despite their prevalence and potential water quality importance, non-perennial rivers are missing from most conceptual and predictive models of aquatic ecosystems^5,6^. This is challenging because biogeochemical processes that predominate during dry phases are not the same as those that are amplified during flowing phases. Thus, there is a critical need to integrate non-perennial rivers directly and holistically into the science, policy, and management of our river systems^7^. Shifts in precipitation and drought due to changing climate and human water extraction are increasing the prevalence and distribution of non-perennial rivers, adding urgency to this scientific need^8^. Below, we identify three barriers and solutions to incorporating biogeochemistry into our understanding of non-perennial rivers. These barriers must be addressed to adequately understand and manage water quality in a changing world.

## Spatial limitation of hydrologic data

The first major barrier is the *spatial limitation of hydrologic data* collected from non-perennial rivers. This barrier results in a large gap in data available to understand non-perennial rivers, which is due to bias in where we locate hydrologic monitoring infrastructure^9^. Gaging networks are disproportionately placed along large, high order perennial rivers, and biased toward temperate regions with higher annual precipitation. Gage networks miss the lower order non-perennial rivers that drain to such gages^9^. The solution to this data gap is clear: there is a pressing need for a more even distribution of monitoring gages. Although non-perennial rivers occur in practically every biome^1^, non-perennial river research to date is largely concentrated in semi-arid, arid, or xeric climates^10,11^ due to greater research and management focus in dry climates. This makes scaling knowledge from field-based studies to larger areas or different biomes prohibitive. Data collection limitations exist because deployment of gages is expensive and labor-intensive. However, emerging hardware solutions using internet of things (IoT) technology are enabling the construction of low cost gages, while high resolution digital elevation models and planned satellite data products will decrease the need for ground truthing sensor data, reducing labor time. The benefits from increased investment in river gages will be amplified by integrating multi-scale observations of water quality and quantity using new technological advances like remote sensing (e.g., colorimetric water quality sensors and water elevation detection) and increasingly affordable distributed in situ sensor networks (e.g., flow presence sensors, temperature sensors) that allow us to gain a higher resolution understanding of longitudinal dynamics within and across river networks^12^. Additional data collection efforts should adopt open, community-driven, collaborative and standardized operating procedures for the type, frequency, and duration of data collected so as to promote future synthesis.

## Limitation of biogeochemical and microbiome data

The second major barrier is that concomitant *biogeochemical and associated microbiome data are even more limited* than the hydrologic data from non-perennial rivers. This barrier arises because these data are rarely collected alongside hydrology data at existing gages. Biogeochemical data such as dissolved oxygen, nutrients (e.g., nitrogen and phosphorus), or sediment loads are orders of magnitude more limited than already limited hydrological data. Despite their recognized importance, microbial community data are almost entirely constrained to individual studies. Monitoring data largely do not exist, save for scarce public health monitoring of pathogenic taxa. Much of what is known about microbial communities in non-perennial waters is inferred based on environmental or functional (e.g., denitrification) measurements. Incorporating microbial data alongside biogeochemical and hydrologic measurements will yield important insights. The solution is again clear: gages with *both* hydrologic and biogeochemical monitoring equipment should be placed in non-perennial areas of river networks to begin closing this critical data gap. This solution must include both retrofitting existing gages with additional biogeochemical sensors, as well as outfitting ungaged, non-perennial stretches of river networks. However, as a key point of distinction from our first identified barrier, this data gap is not *solely* due to a network bias towards studying mid-sized water bodies; it also arises because existing biogeochemical sensors are not always adaptable between wet and dry conditions, and microbiomes do not have readily available sensors to monitor their shifts in real-time. Therefore, we also need adaptation of existing approaches and methods to work in these hybrid aquatic-terrestrial environments. For example, to study how ecosystem metabolism responds to wetting and drying regimes, sensors that can measure oxygen and carbon dioxide levels in both water and air are necessary. The benefit of this investment in infrastructure and methods development would be an increase in our direct measurements of biogeochemical parameters and microbiome shifts, which would allow us to calculate the cumulative impact of non-perennial rivers on water quality.

## The unknown impact of environmental changes

These first two barriers are compounded by the third, which is that *we do not understand how environmental changes—climate change, land use change, and water extraction—will influence duration and frequency of drying*, and thus the biogeochemistry and microbial ecology of these rivers. Climate change is shifting precipitation and drought patterns in novel ways, which will have a direct impact on stream drying characteristics that will vary by region^8^. This shifting baseline creates new scenarios where rivers that have never gone dry before will become non-perennial. To address this, we now must begin to extrapolate beyond current flow conditions into future scenarios. As a solution, more observational data (addressing barriers 1 and 2) can be coupled with experiments mimicking novel environmental conditions. The results of these experiments can be used to improve the ability of ecosystem models to project how newly non-perennial rivers will respond to a changing climate. This information will reduce uncertainty for managers who need to know how changing patterns of streamflow will impact downstream water quality.

Addressing these three barriers with our proposed solutions will allow us to test hypotheses regarding how flow states will influence the chemistry of exported water, the magnitude of export loads, and the suitability of habitat as an aquatic refuge for organisms when surface water dries (Fig. 1a). Testing these hypotheses is fundamental to building our understanding of how non-perennial streams impact water quality, and extrapolating to how changing climate will affect water quality in the future. Changing moisture conditions in the subsurface between wetting events not only modulates activation and maintenance of surface water flow, but it also shapes resident microbiome composition, material accumulation, and biogeochemical processes. For example, even seemingly “puddled” or “dry” streambeds may be active sources of greenhouse gasses (e.g., carbon dioxide, methane; both produced by microbes) if sediment moisture conditions are adequate (Fig. 1b, c). This in turn can influence nutrient retention and cycling rates of microbial communities, affecting river water quality upon rewetting. Conducting biogeochemical studies using models developed where data are available would allow scientists to better predict how fluxes/loads, processing times, ecosystem resilience, and downstream water quality impacts will change in rivers drying under a changing climate.

## Outlook

Climate change is shifting precipitation patterns across the world, resulting in changes in streamflow. Yet, we do not and cannot predict how these shifts will impact downstream water quality due to limitations in where we collect data, the type and scope of data being collected, and the methods and models we can employ to predict future scenarios. Improving our understanding of the contribution of non-perennial rivers to downstream water quality will enhance modeling efforts, thereby facilitating our ability to predict and manage water quality of both perennial and non-perennial waters that are undergoing rapid environmental change. Thus, expanding the scope of river gage networks to encompass non-perennial systems in all biomes is necessary to fill this knowledge gap. Expanding this network so that biogeochemical and microbial research can be co-located with long-term hydrologic data collection is a critical part of this expansion. To ensure our community can collectively address identified research gaps and management needs, we need new coordinated and collaborative approaches to standardize, manage, and share data^12^. For example, continued support of existing and emerging research coordination networks across the globe (e.g., Dry Rivers Research Coordination Network, Aquatic Intermittency effects on Microbiomes in Streams [AIMS] in the US, DRYvER in France) focused on integrating non-perennial systems into river sciences, management, and policy, are valuable mechanisms to leverage the progress we have made in identifying research needs, generate shared knowledge, and create the community of practice that will carry out needed research directions. These emerging scientific networks spanning Europe, Australia, and the United States have synthesized information on the dominant hydrologic behavior of non-perennial rivers, including their global prevalence^1^, their spatial patterns^13^, and their temporal trajectories^8^. However, our understanding of the nexus of hydrology, biogeochemistry and microbiome shifts has been hindered by the barriers articulated herein. As new approaches are developed to integrate various data streams for modeling activities, a centralized database that can link no-flow observations to biogeochemical measurements and microbial data will be a valuable resource. With thoughtful coordination and communication across our global community, we have the opportunity to greatly advance our understanding of the biogeochemical function of currently and soon-to-be drying rivers.

## References

[1] Messager ML (2021). Global prevalence of non-perennial rivers and streams. Nature.

[2] Sabater S, Timoner X, Borrego C, Acuña V (2016). Stream biofilm responses to flow intermittency: from cells to ecosystems. Front. Environ. Sci..

[3] Romaní, A. M., et al. The biota of intermittent rivers and ephemeral streams: prokaryotes, fungi, and protozoans. In *Intermittent rivers and ephemeral streams* (pp. 161–188) (Academic Press, 2017).

[4] Hale RL, Godsey SE (2019). Dynamic stream network intermittence explains emergent dissolved organic carbon chemostasis in headwaters. Hydrol. Process..

[5] Allen DC (2020). River ecosystem conceptual models and non‐perennial rivers: a critical review. Wiley Interdiscip. Rev. Water.

[6] DelVecchia, A.G., et al. Reconceptualizing the hyporheic zone of non-perennial streams. *Freshw. Sci.*, 10.1086/720071 (2022). 10.1086/720071 PMC9280706 35846249

[7] Leigh C (2016). Ecological research and management of intermittent rivers: an historical review and future directions. Freshw. Biol..

[8] Zipper, S. C., et al. Pervasive changes in stream intermittency across the United States. *Environ. Res. Lett.*, **16**, 084033 (2021).

[9] Krabbenhoft C.A., et al. Assessing placement bias of the global gauge network. *Nat. Sustain.*, 10.1038/s41893-022-00873-0 (2022). 10.1038/s41893-022-00873-0 PMC9534037 36213515

[10] Shumilova O (2019). Simulating rewetting events in intermittent rivers and ephemeral streams: a global analysis of leached nutrients and organic matter. Glob. Change Biol..

[11] Sauquet E (2021). Classification and trends in intermittent river flow regimes in Australia, northwestern Europe and USA: a global perspective. J. Hydrol..

[12] Jaeger KL (2021). Beyond streamflow: call for a national data repository of streamflow presence for streams and rivers in the United States. Water.

[13] Hammond JC (2020). Spatial patterns and drivers of non-perennial flow regimes in the contiguous US. Earth.

